# Cyclooxygenase pathway mediates the inhibition of Na-glutamine co-transporter B0AT1 in rabbit villus cells during chronic intestinal inflammation

**DOI:** 10.1371/journal.pone.0203552

**Published:** 2018-09-07

**Authors:** Subha Arthur, Soudamani Singh, Uma Sundaram

**Affiliations:** Department of Clinical and Translational Sciences, Joan C. Edwards School of Medicine, Marshall University, Medical Center Drive, Huntington, WV, United States of America; Universidade Federal do Rio de Janeiro, BRAZIL

## Abstract

In the mammalian intestine, glutamine assimilation by the absorptive villus cells is mediated by Na-glutamine co-transport, specifically by B0AT1. In a rabbit model of chronic intestinal inflammation, B0AT1 is inhibited secondary to a decrease in the number of co-transporters in the brush border membrane (BBM). This inhibition can be reversed by treatment with a broad-spectrum immune modulator such as glucocorticoid suggesting that immune inflammatory mediators may regulate B0AT1 during chronic intestinal inflammation. Arachidonic acid (AA) metabolites (AAM) are increased during chronic intestinal inflammation. However, whether AAM may regulate B0AT1 during chronic intestinal inflammation is unknown. Treatment of rabbits with ATK, to prevent the release of AAM reversed the inhibition of B0AT1. AAM are products of cyclooxygenase (COX) and/or lipoxygenase (LOX) pathways. Inhibition of COX with piroxicam, therefore reduction of prostaglandin formation in the chronically inflamed intestine, reversed the inhibition of B0AT1 to its normal levels. In contrast, inhibition of LOX with MK886, thus reduction of leukotriene formation during chronic enteritis, did not affect the inhibition of B0AT1. Kinetic studies showed that the mechanism of restoration of B0AT1 by ATK or piroxicam was secondary to the restoration of BBM co-transporter numbers. Western Blot analysis also demonstrated restoration of BBM B0AT1 co-transporter numbers. In conclusion, this study demonstrates that Na-glutamine co-transport mediated by B0AT1 in villus cells is regulated by prostaglandins rather than leukotrienes in the chronically inflamed intestine.

## Introduction

Glutamine, a conditionally essential nutrient, has been proven to have abundant health benefits during pathophysiological conditions [1]. For instance, glutamine supplementation has been shown to improve the health status of patients undergoing post-surgical and post trauma treatment, promoting healing and minimizing complications [2–4]. It has also been shown to have a beneficial effect on the patients’ immune system, antioxidant status, heat shock protein response and wound repair [3, 4]. Further, an increase in glutamine demand for cellular metabolism has been demonstrated in these clinical conditions in various studies [2, 5, 6]

Glutamine is also a vital nutrient for rapidly dividing enterocytes, where in addition to serving as a main source of energy for cellular metabolism, it is also involved in the maintenance of structure and function of intestinal epithelium [3, 4, 6]. The gut mucosal cells are known to have high glutaminase activity consistent with their avid rate of glutamine uptake [7]. It has been also reported that the rate of glutamine uptake almost equals the rate of glucose uptake in the intestinal epithelial cells and has been established to be more important than glucose as an oxidative fuel for enterocytes [8]. In the last few decades, the functional properties and molecular characteristics of glutamine transporters across the plasma membrane of enterocytes have been well documented [3, 6, 9, 10]. Luminal transport of glutamine has been shown to occur predominantly via Na-dependent glutamine co-transport pathways and to a lesser extent by Na-independent processes [3, 4, 6, 11].

Different Na-glutamine co-transporters appear to mediate the assimilation of glutamine in the mammalian intestine. In rabbits, Na-glutamine co-transport was found to be mediated by B0AT1 on the BBM. In contrast, in the crypt cells, it was mediated by SN2. In both cell types, glutamine absorption was a secondary active process dependent on basolateral membrane Na-K-ATPase to provide the favorable trans cellular Na gradient. Further, BBM glutamine uptake was approximately four times greater in villus cells compared to crypt cells [9, 10].

Despite the obvious importance of glutamine for intestinal epithelium, how it may be affected in inflammatory bowel disease (IBD), a condition in which intestinal epithelium is at the center of the pathophysiology of the condition, was only recently reported. In a rabbit model of chronic intestinal inflammation analogous to the human IBD, it was shown that the assimilation of glutamine, mediated through Na-glutamine co-transporter B0AT1 in villus cells, was inhibited [9, 10]. At the cellular level, the inhibition was secondary to a decrease in Na-K-ATPase activity, as well as diminished activity of B0AT1. At the co-transporter level, the mechanism of inhibition of B0AT1 was secondary to a reduction in the co-transporter numbers during chronic intestinal inflammation. Finally, despite what appears to be an attempt at compensation by SN2 stimulation in crypt cells, overall intestinal assimilation of glutamine was significantly impaired in chronic intestinal inflammation [12].

A wide variety of immune-inflammatory mediators are endogenously produced in the chronically inflamed intestine. These may have, at least in part, an effect on transport pathways during chronic intestinal inflammation. We have previously shown in the rabbit model of chronic intestinal inflammation that treatment with a broad-spectrum immune modulator such as glucocorticoid alleviates the alterations of Na-glutamine co-transport in villus cells [13]. While this may suggest the participation of several of the immune inflammatory mediators/pathways activated during inflammation, the identity of the specific immune-inflammatory mediator/pathway that might be responsible for the alterations of B0AT1 during chronic enteritis is unknown.

Eicosanoids are found significantly increased in the intestinal mucosa of patients suffering from IBD. The biosynthesis of eicosanoids requires arachidonic acid (AA) which are released from plasma membrane upon hydrolysis of cell-membrane phospholipids by phospholipase A2 (PLA2) [14]. Upon release, AA is converted into unstable endoperoxide intermediates, followed by the production of prostaglandins (PGs) by the cyclooxygenase (COX) pathway or leukotrienes (LTs) through lipoxygenase (LOX) pathway [15]. PGs and LTs have been implicated in the pathogenesis of a number of inflammatory diseases, including rheumatoid arthritis, asthma, psoriasis, multiple sclerosis, and IBD [15–19]. A growing understanding of the etiology and pathophysiology of IBD has brought out the role of AA and its metabolites in IBD. In the intestinal mucosa from patients with IBD a clear increase in eicosanoids such as prostaglandin E2 (PGE2) and leukotriene D4 (LTD4) have been shown [17, 18, 20, 21]. This suggests that regulation of PLA2 and or AA pathway metabolites could be a therapeutic option for IBD. Considering the importance of AA and its metabolites during inflammation, we hypothesized that inhibition of PLA2 activation by Arachidonyl Trifluoromethyl Ketone (ATK) and inhibition of AA pathway enzymes: COX by piroxicam (PRX) or LOX by MK886, may reverse reduced Na-glutamine co-transport seen during inflammation.

Given this background, the aim of the present study was to identify and delineate the role of AA and subsequently the COX/LOX pathways in the regulation of Na-glutamine co-transporter B0AT1 in the rabbit model of chronic intestinal inflammation.

## Materials and methods

### Animals

New Zealand White male rabbits weighing between 2.0–2.2 kg were procured form Charles River Laboratories (PA, USA). All the rabbits were housed in individual stainless steel cages in animal house facility at 22±2 ^0^C with 50–70% humidity and 12h light and dark cycle. All the rabbits had free access to rabbit diet (5321-Laboratory Rabbit Diet; PMI nutrition International, St. Louis, MO, USA) and water, and had a week of acclimatization before the study. All the components of this animal study including animal handling procedures, treatments and euthanization were approved by the ethics committee of Marshall University’s Instititional Animal Care and Use Committee (Protocol Reference number 668). All the animal procedures and treatments in this study were carried out in accordance with the regulations of this committee.

### Initiation of chronic inflammation and drug treatment

After 1 week of acclimatization, all the animals were divided in eight groups, each group containing 4 animals. Group-1 included control rabbits that were treated intramuscularly with saline. Group-2 included rabbits with induced chronic intestinal inflammation. Chronic inflammation was induced in these rabbits with an intragastric inoculation of *Eimeria magna* oocytes, as previously reported [22, 23]. In Group-3, rabbits were treated with 3 mg/kg of ATK, an analog of AA, while animals in group-4 included rabbits with chronically inflamed intestine treated with ATK. Group-5 had normal rabbits treated with 10 mg/kg of PRX, an inhibitor of cyclooxygenase pathway. Group-6 included rabbits induced for chronic enteritis and treated with PRX. Group-7 had normal rabbits treated with 0.5 mg/kg of MK886, an inhibitor of lipoxygenase pathway. Group-8 contained rabbits with chronic intestinal inflammation that were treated with MK886. All the treatments were performed as intramuscular injections on days 12 and 13, post induction of inflammation and corresponding days for normal rabbits. The inflamed animals and the animals in the treatment groups were monitored every 24 hours for any adverse clinical affects during the course of the study. No adverse clinical effects on the animals were recorded in the present study. All the animals were euthanized on day 14, as per IACUC guidelines.

### Cell isolation

Calcium chelation technique was used for the isolation of villus cells from the rabbit ileum, as previously described [10, 12]. Briefly, a 3-ft section of ileum was filled and incubated in cell isolation buffer (0.15mM EDTA, 112 mM NaCl, 25mM NaHCO3, 2.4 mM K2HPO4, 0.4mM KH2PO4, 2.5 mM L-glutamine, 0.5 mM β-hydroxybutyrate, and 0.5 mM dithiothreitol; gassed with 95% O2, and 5% CO2, pH 7.4, at 37°C) for 3 min and was gently palpitated for another 3 min to facilitate cell dispersion. The buffer was then drained out from the ileal section, and the suspension was centrifuged at 100 g for 3 min. The cells were then flash frozen in liquid nitrogen and stored at −80°C until further use.

### Na-K-ATPase measurement

Cellular homogenates were used to assess Na-K-ATPase activity levels which was measured using a previously described protocol [24]. Na-K-ATPase enzyme-specific activity was expressed as nanomoles of inorganic phosphate released per milligram protein per minute.

### Uptake studies in villus cells

Na-glutamine uptakes in intact villus cells were done using previously described protocol [9, 10, 12]. Villus cells (100 mg wet wt.) were washed and suspended in HEPES buffer (0.2 mM glutamine, 4.5 mM KCl, 1.2 mM KH2PO4, 1.0 mM MgSO4, 1.25 mM CaCl2, 20 mM HEPES), with either 130 mM sodium chloride or choline chloride. Uptake experiment with these cell suspensions were performed as described previously.

### BBMV preparation and uptake studies

Villus BBMV were prepared by CaCl2 precipitation and differential centrifugation as previously described [9, 10, 12]. Rapid filtration technique was employed to do the uptake experiment [12]. In brief, 5 μL of BBMV suspended in vesicle medium (100 mM choline chloride, 0.10 mM MgSO4, 50 mM HEPES-Tris (pH 7.5), 50 mM mannitol, 50 mM KCl) was incubated in 95 μL of reaction medium (50 mM HEPES-Tris buffer; pH 7.5, 0.2 mM glutamine, 20 μCi ^3^H-glutamine, 0.10 mM MgSO4, 50 mM KCl, 50 mM mannitol, 100 mM of either NaCl or choline chloride) and at 30 second time point the uptake was arrested by mixing with ice-cold stop solution (50 mM HEPES-Tris buffer (pH 7.5), 0.10 mM MgSO4, 75 mM KCl, and 100 mM choline chloride). 0.45 μm Millipore (HAWP) filter was used to filter the stopped reaction mixture and radioactivity was determined in Beckman Coulter LS6500 scintillation counter. For kinetic studies, Na-dependent glutamine uptake was performed at 6 seconds by varying the concentration of extra vesicular glutamine (0.2, 0.5, 1, 5, 10, 25, 50, 75 and 100 mM). Data derived from these experiments were analyzed with GraphPad Prism 4 (San Diego, CA) for Michaelis-Menten kinetics using a non-linear regression data analysis to derive kinetic parameters.

### Western blot analysis

BBM protein extract solubilized in a RIPA lysis buffer (Santa Cruz Biotechnology, Inc. Santa Cruz, CA) was used for the Western Blot analyses. Equal amount of proteins were denatured in sodium dodecyl sulfate sample buffer (20 mM Tris pH 7, 12% glycerol, 2% SDS, 0.01% bromophenol blue, and freshly added 1 mM Dithiothreitol) and separated by electrophoresis on a 8% Gel (Bio-Rad Laboratories, Hercules, CA). Proteins on the gel were transferred to a polyvinylidene membrane which was blocked with 5% bovine serum albumin in TBS (20 mM Tris, pH 7.5, 150 mM NaCl) with 0.1% Tween-20 and then incubated overnight at 4°C with goat polyclonal antibody against B0AT1 followed by incubation with anti-goat IgG conjugated to horseradish peroxidase (Jackson Immunoresearch Laboratories, West Grove, PA) for an hour at room temperature. The primary antibody was obtained through the custom antibody services provided by Invitrogen. ECL reagent for Western blotting detection (RapidStep™ ECL Reagent, Millipore) was used to detect the immobilized protein. The resultant chemiluminescence was detected using biomax film (Kodak, Rochester, NY) and the intensity of the bands was analyzed with FluorChem^TM^ instrument (Alpha Innotech, San Leandro, CA).

### Statistical analysis

Results in all figures are shown as mean ± SEM that were calculated with GraphPad InStat program. All individual uptakes were done in quadruplicate. The ‘n’ number for any set of uptake experiments or western blot analysis refers to vesicle or isolated cell preparations from different animals. Student’s t-test was used for statistical analysis and *p<0*.*05* was considered significant.

## Results

### Effect of ATK on Na-glutamine co-transport in the intact villus cells

**Fig 1:**
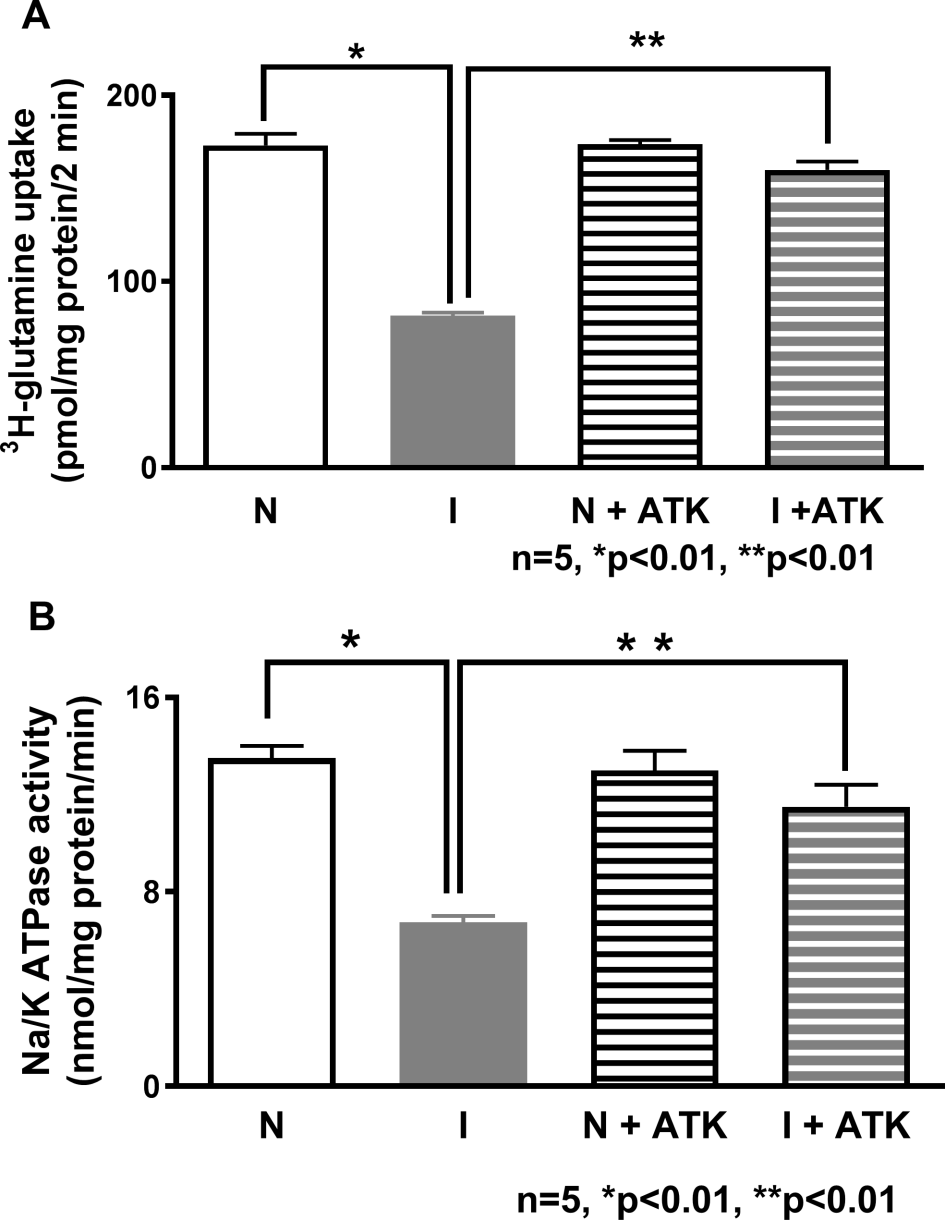
Effect of ATK on Na-glutamine co-transport and Na-K-ATPase activity in intact villus cells. (A) Na-dependent glutamine uptake was significantly decreased in villus cells from the chronically inflamed intestine and in vivo treatment with ATK reversed this inhibition. ATK had no effect on Na-glutamine co-transport in villus cells from the normal intestine (B) Na-K-ATPase activity that was significantly reduced in the villus cells during chronic enteritis was reversed by ATK. Na-K-ATPase activity in ATK treated normal intestine remained unaffected.

Na-glutamine co-transport, which was significantly reduced in the villus cells from the chronically inflamed intestine, was reversed to near normal levels by *in vivo* ATK treatment (Fig 1A). ATK did not have any effect in the villus cells from the normal intestine (173±6 pmol/mg protein•2 min in normal; 82±2 in inflamed; and 159±4.5 in inflamed+ATK; *p<0*.*05*, n = 4). These data indicate that a metabolite of AA pathway is involved in the regulation of Na-glutamine co-transport in inflamed villus cells.

### Effect of ATK on Na-K-ATPase Activity

Na-dependent co-transporters require Na-K-ATPase to function to its optimal levels, as it provides the favorable trans cellular Na gradient. Previous studies have shown that Na-K-ATPase is significantly inhibited in the villus cells during chronic intestinal inflammation [12]. In the present study, there was no difference in Na-K-ATPase activity in the villus cells from normal and normal+ATK (Fig 1B: 12.2±1.7 nmol/mg protein•min in normal and 13±0.8 in normal+ATK; n = 4). The significant decrease in Na-K-ATPase activity seen during chronic intestinal inflammation was restored by ATK treatment (Fig 1B: 3.1±0.5 nmol/mg protein•min in inflamed and 11.5±0.9 in Inflamed+ATK; *p<0*.*05*, n = 4). These data indicate that diminished villus trans cellular Na gradient, is at least partially responsible for the diminished Na-glutamine co-transport in the chronically inflamed intestine, but is reversible by ATK treatment.

### Effect of ATK on Na-glutamine Co-transport in the villus cell BBMV

**Fig 2:**
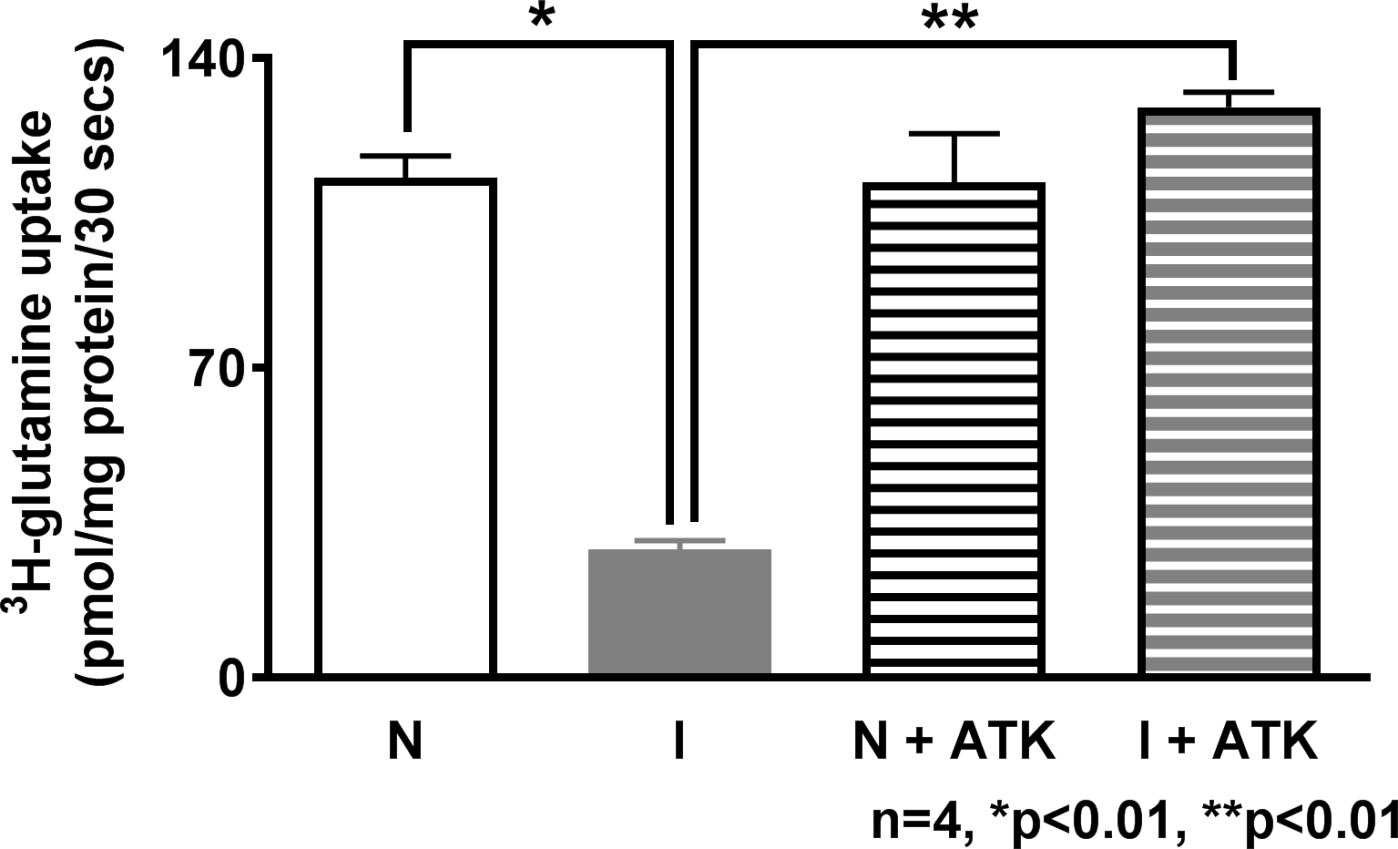
Effect of ATK on Na-glutamine co-transport in villus cell BBMV. Na-dependent glutamine uptake was significantly decreased in villus cell BBMV from the chronically inflamed intestine and this inhibition was reversed with *in vivo* ATK treatment. Na-glutamine co-transport was not affected in the normal intestine.

To determine the effect of ATK on Na-glutamine co-transport at the co-transporter level in the BBM, uptake studies were performed in BBM vesicles (BBMV) prepared from villus cells. Na-glutamine co-transport was significantly inhibited in the villus cell BBMV from the chronically inflamed intestine (Fig 2: Na-glutamine co-transport uptake in BBMV from the normal intestine was 108.2±4.9 pmol/mg protein•30 sec and 37.6±0.4 in the chronically inflamed intestine; n = 4, p<0.05). When chronically inflamed rabbits were treated with ATK there was a significant reversal of inhibition of Na-glutamine co-transport to near normal levels (129.8±3.3 pmol/mg protein•30 sec in inflamed+ATK; n = 4, p<0.05). In normal rabbits, ATK treatment had no effect on Na-glutamine co-transport (112.2±11 pmol/mg protein•30 sec). These data indicate that AAM regulate Na-glutamine co-transport in the chronically inflamed rabbit intestine.

### Effect of ATK on kinetics of BBM Na-glutamine co-transport

**Fig 3:**
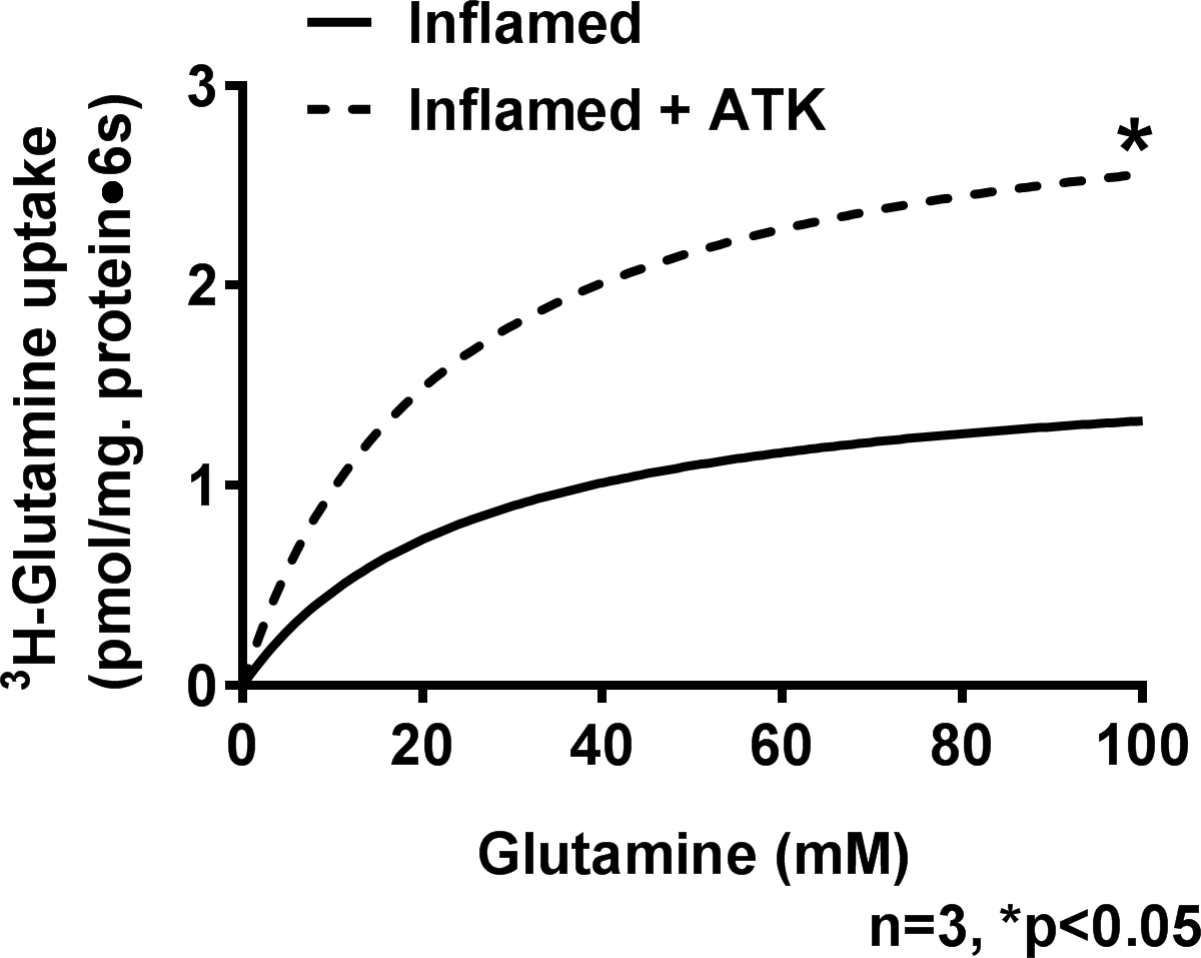
A representative graph of kinetics of glutamine uptake in villus BBMV. As the concentration of extravesicular glutamine was increased the uptake of glutamine also increased and became saturated in both inflamed and inflamed+ATK, as shown in the above figure.

Kinetic studies were performed to determine the mechanisms of reversal of inhibition of Na-glutamine co-transport by ATK. Kinetics for Na-dependent glutamine uptake was performed as a function of increasing concentrations of extra vesicular glutamine at a constant time of six seconds. As the concentration of extra vesicular glutamine was increased, glutamine uptake became stimulated, and subsequently saturated in all conditions (Fig 3). The numbers obtained with kinetics experiments were analyzed using GraphPad Prism 4 (San Diego, CA) to determine the kinetic parameters of BBM Na-glutamine co-transport in all experimental conditions.

In the chronically inflamed intestine, Na-glutamine co-transport was inhibited in villus cells secondary to a decrease in the maximal rate of glutamine uptake (Vmax) without a change in the affinity (Km) of the co-transporter for glutamine. Treatment of rabbits with chronic enteritis with ATK reversed the inhibition by restoring the Vmax or number of co-transporters, without a change in the affinity of the co-transporter for its substrate (Table 1). These data indicate that ATK reverses the inhibition of Na-glutamine co-transport in the chronically inflamed intestine by restoring BBM co-transporter numbers.

**Table 1:** Kinetic parameters of Na-glutamine co-transport in AKT treated villus cell BBMV.

	Vmax (nmol/mg protein• 6 sec)	Km (mM)
**Control**	**3.7 ± 0.5**	**24.6 ± 1.8**
**Inflamed**	1.8 ± 0.1*	**26.6± 2.0**
**Inflamed + ATK**	3.2 ± 0.1*	**22.5 ± 0.2**

In villus cells, the maximal rate of uptake (Vmax) of Na-glutamine co-transport which was significantly diminished in chronically inflamed intestine was reversed by AKT treatment (n = 3, *p<0.*05*). However, the affinity was unchanged in all conditions.

### Effect of AA pathway inhibition by ATK on B0AT1 protein expression in villus cell BBM

**Fig 4:**
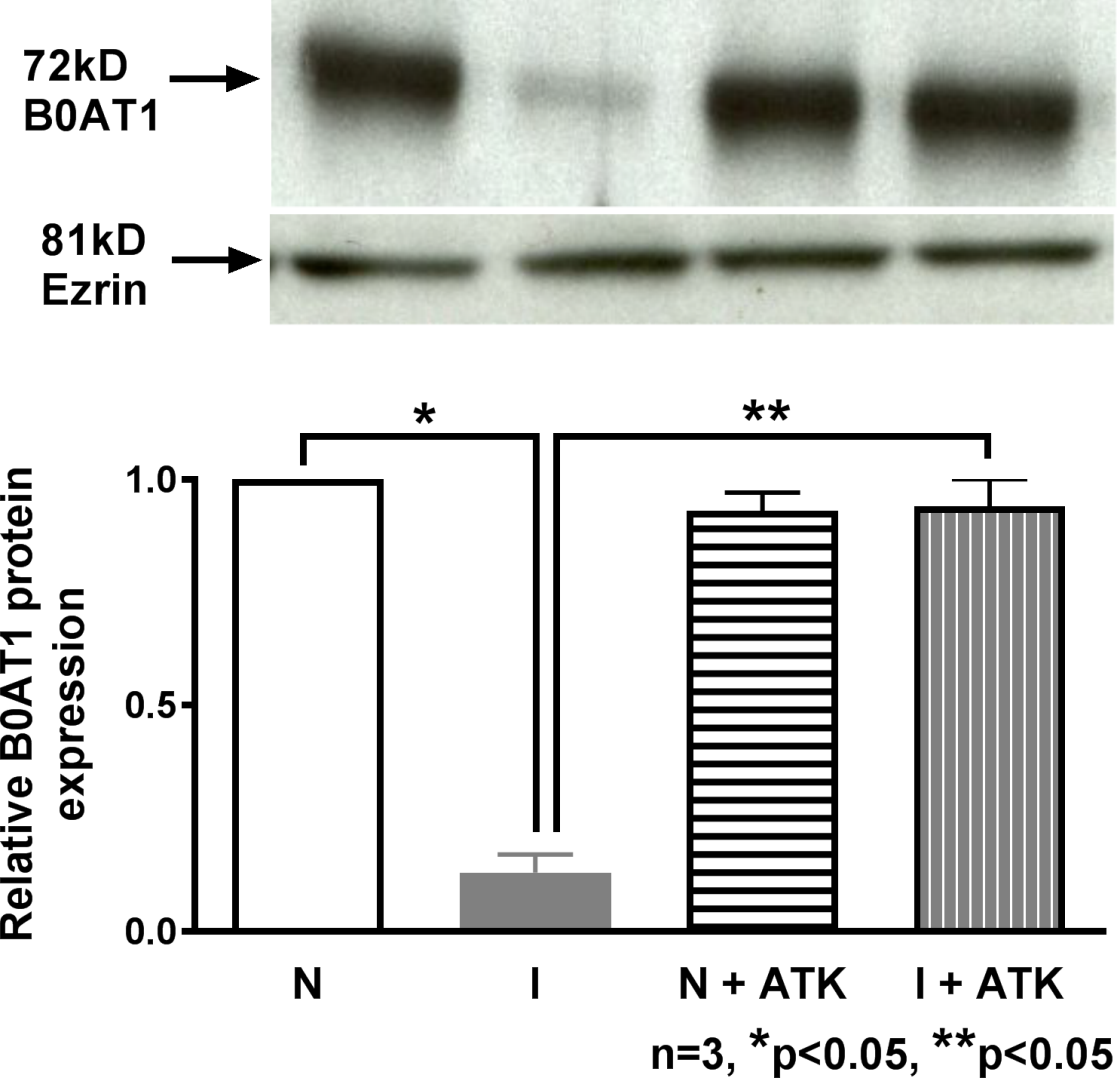
B0AT1 protein expression in AKT treated villus cell BBM. A representative Western blot of BBM B0AT1 protein levels is shown in the upper panel. In the lower panel densitometric quantitation is shown. The relative protein expression of B0AT1 that was significantly reduced during chronic intestinal inflammation was restored to near-normal levels by AKT treatment and B0AT1 levels were unaffected in the normal intestine.

We have previously shown that B0AT1 is the major Na-dependent glutamine co-transporter in the BBM of rabbit villus cells [12]. Therefore, in the present study immunoreactive protein levels of B0AT1 was determined in the villus cell BBM from all experimental conditions. Fig 4. shows that B0AT1 protein level was significantly reduced in villus cell BBM during chronic enteritis. ATK treatment restored B0AT1 immunoreactive levels in the villus cell BBM from the chronically inflamed intestine. ATK treatment did not change B0AT1 protein levels in normal. These results in conjunction with kinetic parameters above indicate that ATK reverses the inhibition of B0AT1 during chronic enteritis by restoring BBM B0AT1 co-transporter numbers.

### Effect of COX and/or LOX pathway inhibition on Na-glutamine co-transport

**Fig 5:**
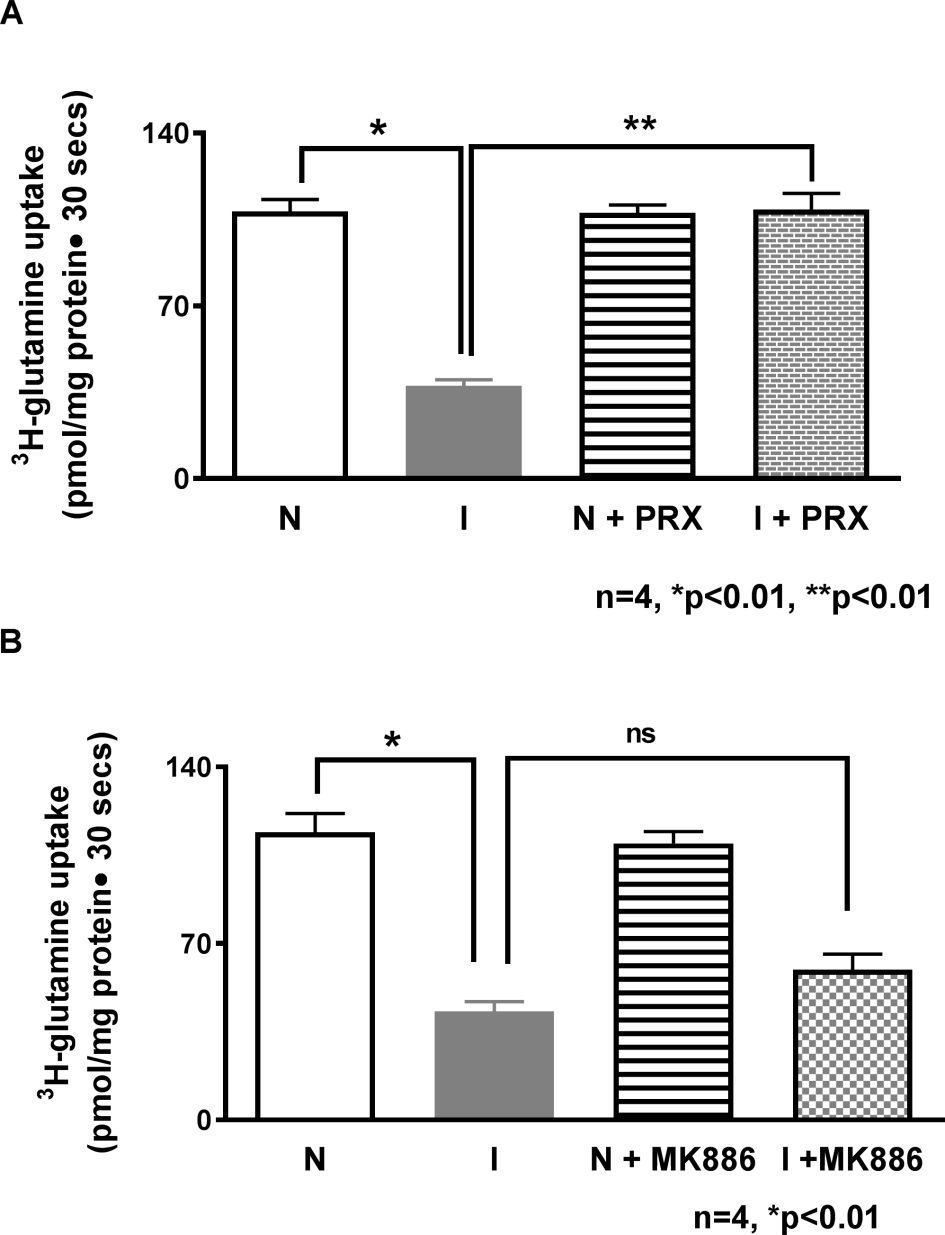
Effect of inhibition of COX and LOX pathways on villus BBM Na-glutamine co-Transport. (A) Effect of piroxicam on Na-glutamine co-transport in villus cells BBMV. Na-dependent glutamine uptake was significantly decreased in villus cell BBMV from the chronically inflamed intestine and this inhibition was reversed with *in vivo* piroxicam treatment. Na-glutamine co-transport was not affected in the normal intestine. (B) Effect of MK886 on Na-glutamine co-transport in villus cells BBMV. Na-dependent glutamine uptake which was significantly decreased in villus cell BBMV from the chronically inflamed intestine and this inhibition was not affected by *in vivo* MK886 treatment. Na-glutamine co-transport was not affected in the normal intestine.

Since the COX and/or LOX pathway may produce AAM, rabbits were treated with either COX or LOX inhibitors to determine inflammation mediated specific regulation of B0AT1. Treatment of rabbits with chronic enteritis with COX inhibitor piroxicam, reversed the inhibition of Na-glutamine co-transport in villus cell BBMV (Fig 5A: 109.9±3.2 pmol/mg protein•30 sec in inflamed+PRX; n = 4, p<0.05). PRX had no effect in the normal intestine (Fig 5A: 109.7±4.7 pmol/mg protein•30 sec; n = 4). In contrast, inhibition of the LOX pathway with MK886 treatment failed to reverse the inhibition of Na-glutamine co-transport in villus cell BBMV from the chronically inflamed intestine (Fig 5B: 59.9±4.3 pmol/mg protein•30 sec; n = 4). These data indicate that AAM regulates Na-glutamine co-transport in the chronically inflamed intestine, specifically through COX, but not the LOX pathway.

### Effect of COX pathway inhibition on kinetic parameters of Na-glutamine co-transport

**Fig 6:**
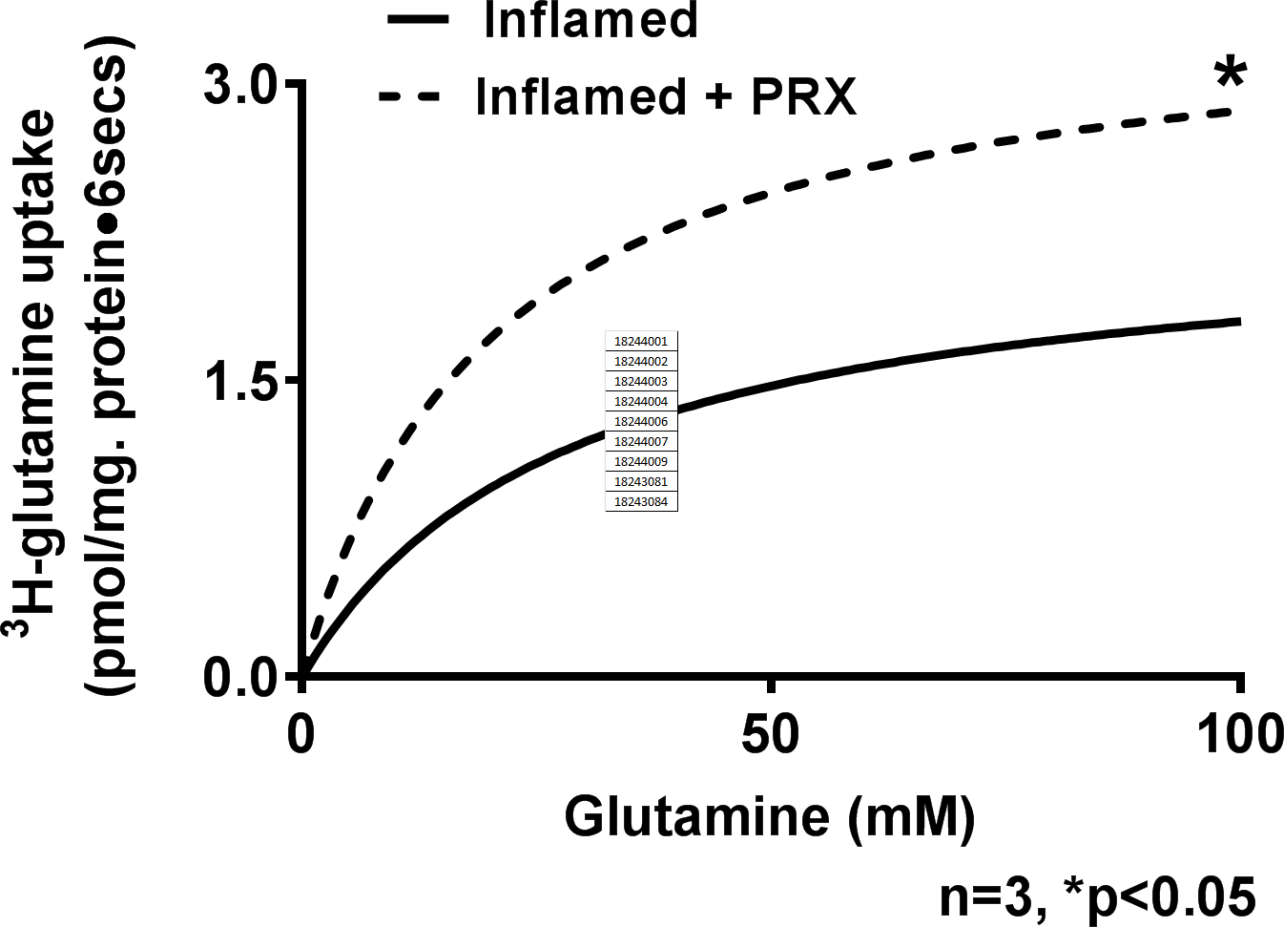
A representative graph of kinetics of glutamine uptake in inflamed and inflamed+PRX villus BBMV. The kinetic parameters derived from an n = 3 of such experiments are shown in Table 2.

Kinetic studies with piroxicam (Fig 6) showed that it reversed the inhibition of Na-glutamine co-transport in villus cells by restoring the Vmax, and without affecting the affinity of the co-transporter (Table 2). Thus, the mechanism of reversal of Na-glutamine co-transport inhibition by PRX was secondary to restoration of co-transporter number (Vmax) with no change in its affinity (Km) for its substrate glutamine.

**Table 2:** Kinetic parameters of Na-glutamine co-transport in PRX treated villus cell BBMV.

	Vmax (nmol/mg protein•6 sec)	Km (mM)
**Control**	**3.4 ± 0.1**	**24 ± 0.6**
**Inflamed**	1.9 ± 0.2*	**27.2± 1.2**
**Inflamed + PRX**	3.3 ± 0.1*	**23.3 ± 1.6**

PRX treatment reversed the maximal rate of uptake (Vmax) of Na-glutamine co-transport which was significantly diminished in chronically inflamed intestine (n = 3, *p<0.*05*). The affinity of the BBM Na-glutamine co-transporters remained unchanged in all conditions.

### Effect of COX pathway inhibition on B0AT1 protein expression in villus cell BBM

**Fig 7:**
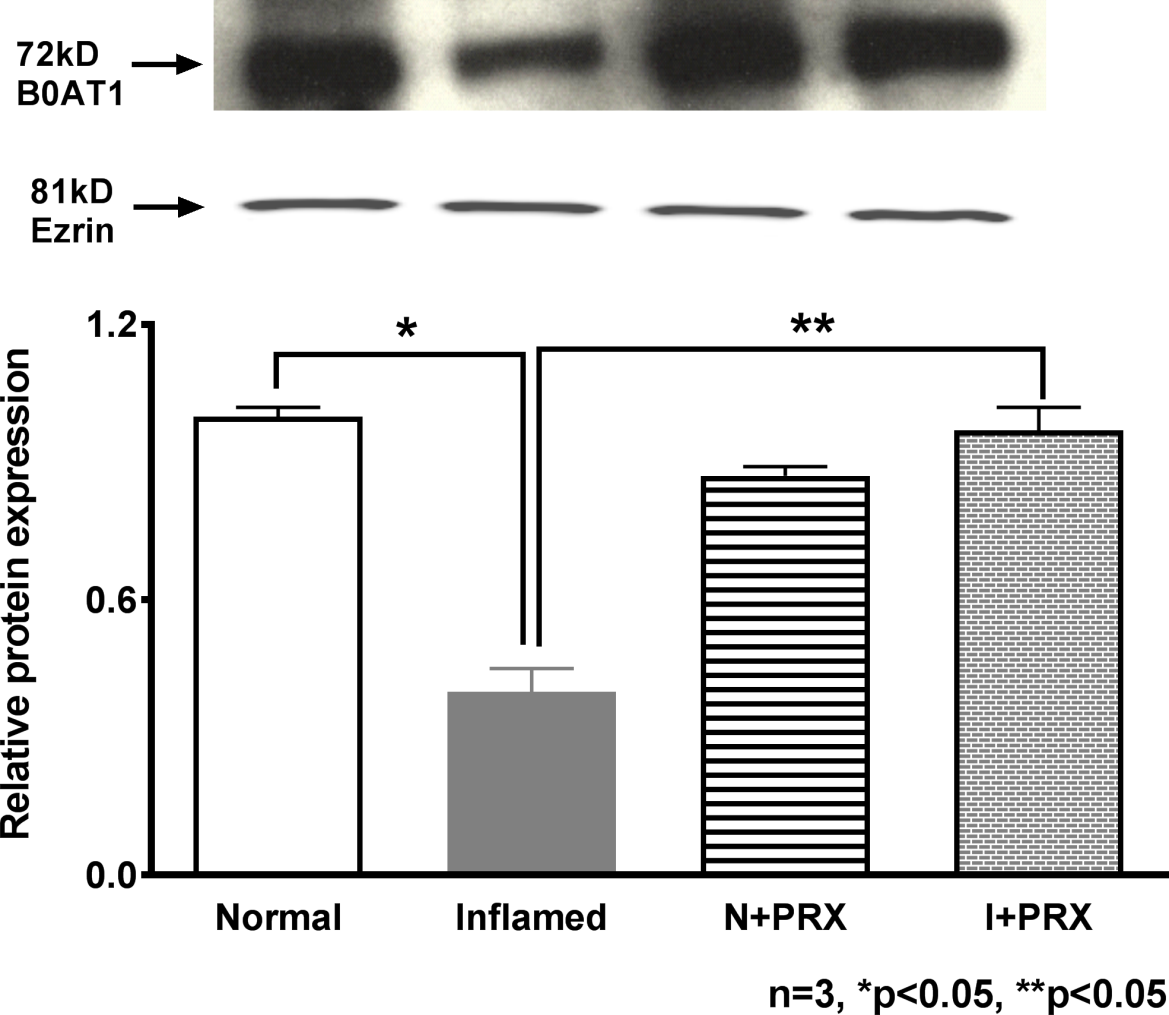
B0AT1 protein expression in PRX treated villus cell BBM. A representative blot of B0AT1 protein levels is shown in the upper panel. In the lower panel, densitometric quantitation is shown. The relative protein expression of B0AT1 that was reduced during chronic intestinal inflammation was restored to near-normal levels by PRX treatment and B0AT1 levels remained unaffected in the normal intestine.

Analysis of the immunoblot for B0AT1 protein in the villus cell BBMV is shown in Fig 7. B0AT1 protein levels were significantly inhibited during chronic enteritis. PRX treatment restored B0AT1 immunoreactive levels in the villus cell BBM from the chronically inflamed intestine. Piroxicam did not have any effect on the B0AT1 protein levels in normal animals. These data, in concert with the kinetic results above show that the mechanism of piroxicam mediated reversal of Na-dependent glutamine co-transport is through the restoration of the B0AT1 numbers in the BBM of villus cells from the chronically inflamed intestine.

## Discussion

This study demonstrates that the inhibition of B0AT1 in villus cells during chronic intestinal inflammation is mediated by arachidonic acid metabolites. More specifically, AAM formed by the cyclooxygenase and not the lipoxygenase pathway. Pharmacological inhibition of both, arachidonic acid release as well as the production of AAM by the COX pathway reversed the inhibition of B0AT1 in the villus BBM by the restoration of diminished B0AT1 co-transporter numbers. Thus, this study demonstrated that arachidonic acid metabolites, specifically prostaglandins, regulate Na-glutamine co-transport in villus cells in the chronically inflamed intestine.

B0AT1 is the predominant Na-glutamine co-transporter in the BBM of villus cells responsible for glutamine assimilation in the rabbit intestine. It is inhibited during chronic intestinal inflammation likely by immune inflammatory mediators known to be up regulated in the mucosa. Consistent with this hypothesis was the previous demonstration that treatment with a broad-spectrum immune modulator, namely corticosteroid methylprednisolone reversed the inhibition of B0AT1 [13]. Since a wide array of complex immune pathways participate in inflammatory processes, it becomes important to determine the specific immune inflammatory pathway/mediators that might be involved in the regulation of absorption of the important nutrient glutamine, mediated by B0AT1. One such immune inflammatory pathway that has been shown to play a significant role in the pathogenesis and progression of disease is AA pathway and its metabolites [14, 17–21]. However, how this pathway might affect Na-glutamine co-transporter B0AT1 in the chronically inflamed intestine was previously unknown. This study demonstrated that the AAM produced by the COX, not the LOX pathway is responsible for the inhibition of villus cell B0AT1 during chronic enteritis [25].

The participation of COX, and not LOX pathway in the regulation of villus specific B0AT1 is in accordance with the previous report by Singer *et al*., [25] where it was shown that COX-1 and COX-2 expression was present only in villus but not in crypt cells. COX-1 is known to be constitutively expressed in normal and inflamed villus cells [26]. Whereas, COX-2 expression is increased during chronic intestinal inflammation [26]. COX-2 expression was also shown in the surface of colonic epithelial cells from ulcerative colitis and Crohn’s colitis but not in normal colon or normal ileum [25, 26]. Handel and Nielsen [26], by using reverse transcription-polymerase chain reaction, found the presence of COX-2 mRNA in rectal biopsy specimen obtained from patients with ulcerative colitis and Crohn’s disease but not in normal controls [25, 27]. These findings suggest that the increased levels of PGs in the inflamed intestinal tissue results from increased expression of COX-2 but not COX-1 [25]. Therefore, it seems likely that reversal of inhibition of Na-glutamine co-transport mediated by B0AT1 in villus cells might be due to inhibition of COX-2 by PRX. The identity of the specific prostaglandin that might be involved in the regulation of B0AT1 during chronic intestinal inflammation needs to be elucidated by further investigation.

It becomes important to identify the specific immune inflammatory mediator that might regulate glutamine absorption because glutamine plays a vital role in the maintenance of villus cell metabolism, structure, and function in normal physiological conditions [8]. The importance of glutamine in the gut can be highlighted further by the fact that glutamine enriched nutrition provides protection against atrophy of the villus cells in rats [28–30]. Glutamine is also known to increase villus cell height [29] and instill the ability to accelerate mucosal regeneration after starvation in the gut [8]. Glutamine has been not only used successfully in the treatment of peptic ulcers [31] but it has also been shown to protect against aspirin-induced gastric ulceration [28]. The importance of the restoration of glutamine absorptive mechanism and therefore the intestinal health in the inflamed intestine could be validated by a study where infusion of the enzyme glutaminase systemically resulted in the development of diarrhea, villus atrophy, mucosal ulcerations, and intestinal necrosis in several animal species [31]. It has also been established in humans that the provision of glutamine-supplemented nutritional support is an important adjunct to the therapy of patients with an intestinal mucosal injury attained secondary to chemotherapy and radiation therapy [32]. Also, glutamine is known to provide small bowel mucosal protection, decrease translocation of luminal bacteria [31], accelerate healing of the gut mucosa, and improve survival [33].

**Fig 8:**
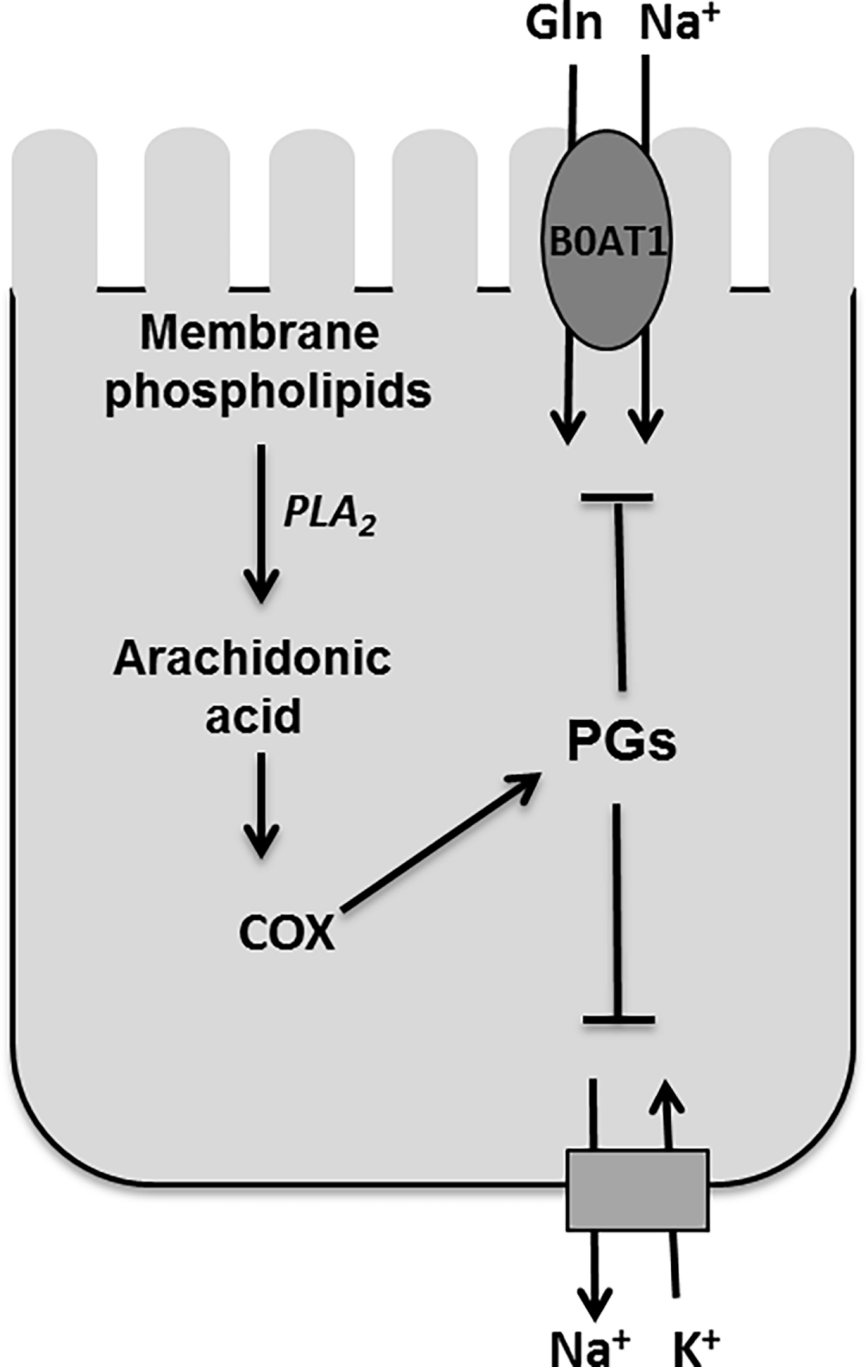
Cyclooxygenase pathway mediated inhibition of Na-glutamine co-transporter B0AT1 in rabbit villus cells during chronic intestinal inflammation. Na-glutamine co-transporter B0AT1 is regulated through arachidonic acid pathway, specifically COX pathway, in mammalian villus cells during chronic intestinal inflammation.

In conclusion, Na-dependent glutamine co-transporter B0AT1 that was inhibited during chronic intestinal inflammation in villus BBM was restored to normal by both ATK and piroxicam treatments while MK886 did not have any effect. These observations tempt to speculate that downstream regulation of BBM villus Na-glutamine co-transport might be mediated through PGE2 and not by LTD4 (Fig 8). The mechanisms of ATK and PRX mediated restoration of function whereby reversing the same mechanisms that resulted in the original alteration, specifically, diminished co-transporter numbers in villus during chronic intestinal inflammation. These observations indicate that arachidonic acid likely functions as an upstream broad-spectrum immune modulator responsible for the unique regulation of B0AT1 in villus during chronic intestinal inflammation. More specifically, COX pathway and likely one of its metabolites are responsible for the regulation of B0AT1 in villus BBM during chronic enteritis.
