# Correction: Cyclooxygenase pathway mediates the inhibition of Na-glutamine co-transporter B0AT1 in rabbit villus cells during chronic intestinal inflammation

**DOI:** 10.1371/journal.pone.0294387

**Published:** 2023-11-09

**Authors:** Subha Arthur, Soudamani Singh, Uma Sundaram, The PLOS ONE Editors

Following publication of this article [1], the authors contacted PLOS to request a Correction of Fig 4. They self-reported to PLOS that while using a common figure template, the representative Ezrin loading control panel from Figure 5 of [2] was inadvertently used in Fig 4 of this manuscript [1]. A corrected version of Fig 4 is provided here. The authors provided image data for the B0AT1 and Ezrin blots in the corrected figure, as well as B0AT1 and Ezrin blots from independent replicates (S1 and S2 Files). They also provided quantitative data in support of the graph in Fig 4 (S3 File).

During the editorial assessment of the image data, concerns were raised that the B0AT1 results for experiments 2 & 3 appear qualitatively different than the results reported in the published figure (see S1 and S2 Files provided below). In addition, the editors noted that the quantitative densitometry data provided for repeat experiments 2 and 3 appear to convey different results than the corresponding image data.

The authors stand by the published results and the data in S1–S3 Files. They stated that variability across replicates was expected due to natural variation across individual animals. Regarding the quantitative data, the authors stated that they reanalyzed the original blots using the same equipment and software as was used in the original analysis, and that this reanalysis supported the published results. In addition, the authors stated that the quantitative data were analyzed by a biostatistician at their institute, who verified the statistical significance of the data in the Fig 4.

The *PLOS ONE* Editors remain concerned about inconsistencies across the dataset; these issues were not resolved by the authors’ responses. However, per the editorial assessment the data provided for replicate experiments 1, 4, and 5, appear to support the published findings.

This Correction resolves the image duplication issue discussed above, provides the primary data for the experiment reported in Fig 4, and conveys the outcome of the editorial assessment. Readers are advised to review the primary data for Fig 4 and interpret the article’s findings accordingly.

The dataset originally posted at the Open Science Framework (OSF) had mean ± sem and not individual-level data. The individual-level data has now been added as an amendment to the OSF deposition (https://osf.io/hmxy2/?view_only=f99b844e599d44e19b0e1a36ba9e6aab).

**Fig 4:**
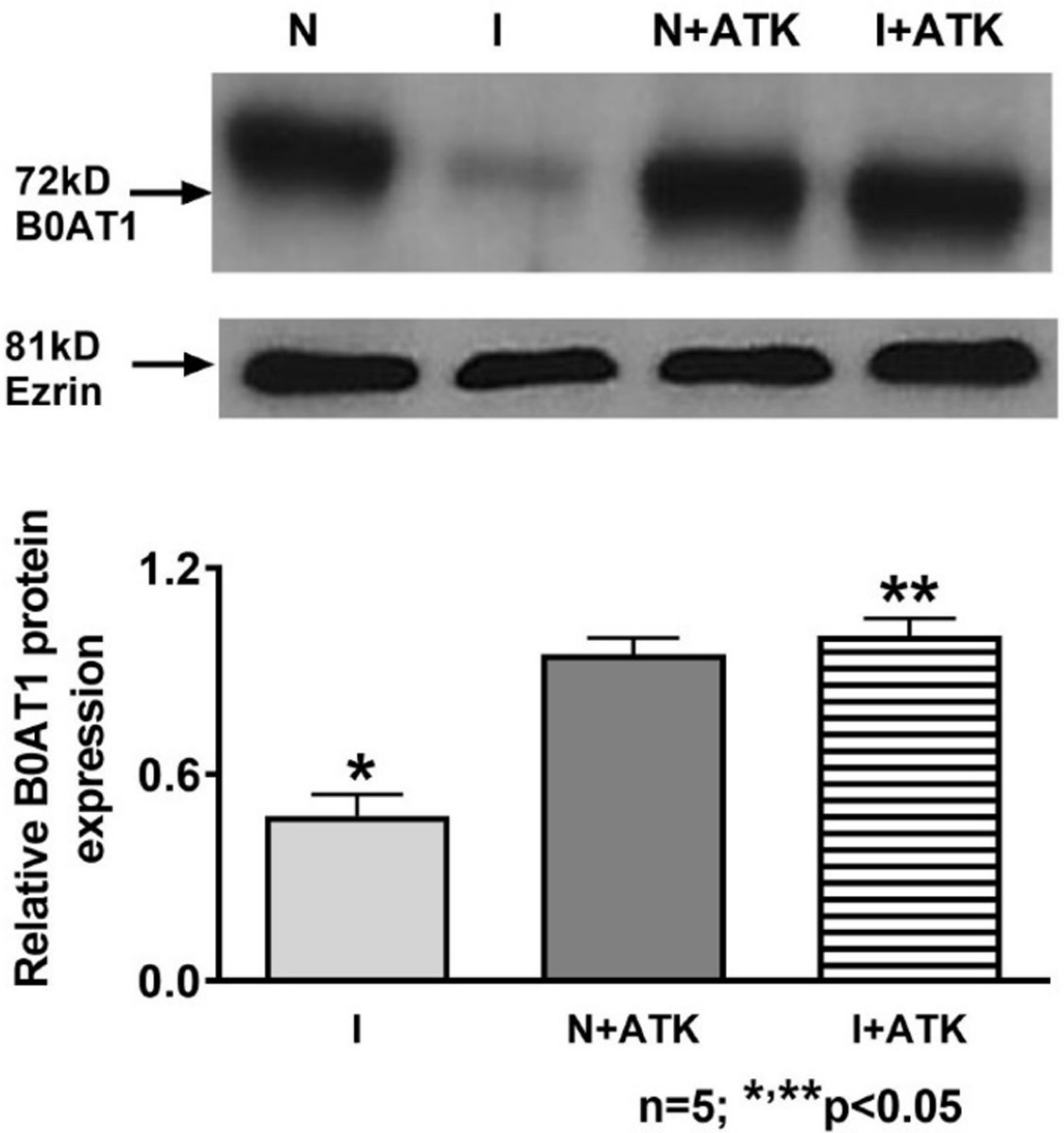
B0AT1 protein expression in AKT treated villus cell BBM. A representative Western blot of BBM B0AT1 protein levels and its related ezrin are shown in the upper panel. The densitometric quantitation of the B0AT1 and ezrin bands were done with FluorChem SP software integrated with the Alpha Innotech FluorChem^TM^ instrument. The Integrated Density values (IDV) obtained for each experiment were normalized by dividing the IDV value of B0AT1 by the IDV value of its related ezrin band. The values thus obtained for I, N+ATK, and I+ATK were then calculated as relative to N, which was set to 1. Student t-test analysis of data showed that inflamed (I) was significantly different (lower) from normal and inflamed treated with ATK was significantly different (higher) from inflamed.

## Supporting information

S1 File Original unadjusted and uncropped image underlying the updated version of Fig 4, and data for B0AT1-Experiments 2 and 3. (PDF)

S2 File Original unadjusted and uncropped image underlying the repeat data for B0AT1-Experiments 4 and 5. (PDF)

S3 File Quantitative data underlying Fig 4 repeat samples 1–5. (PDF)
