# Supplementary material for: Correction: Cyclooxygenase pathway mediates the inhibition of Na-glutamine co-transporter B0AT1 in rabbit villus cells during chronic intestinal inflammation
Source: PLoS One. 2023 Nov 9;18(11):e0294387. doi: 10.1371/journal.pone.0294387 (PMC10635497; doi:10.1371/journal.pone.0294387)

B0AT1-Experiment 1 (representative Blot in Figure 4)

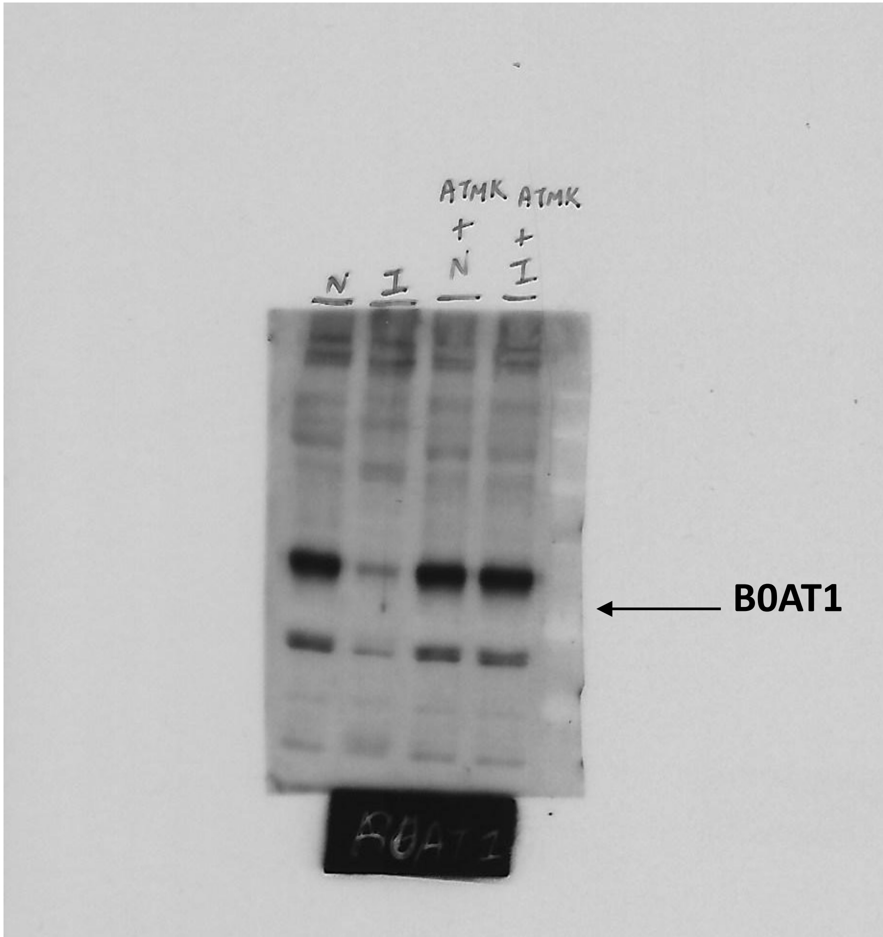

Ezrin-Experiment 1 (Representative blot in Figure 4)

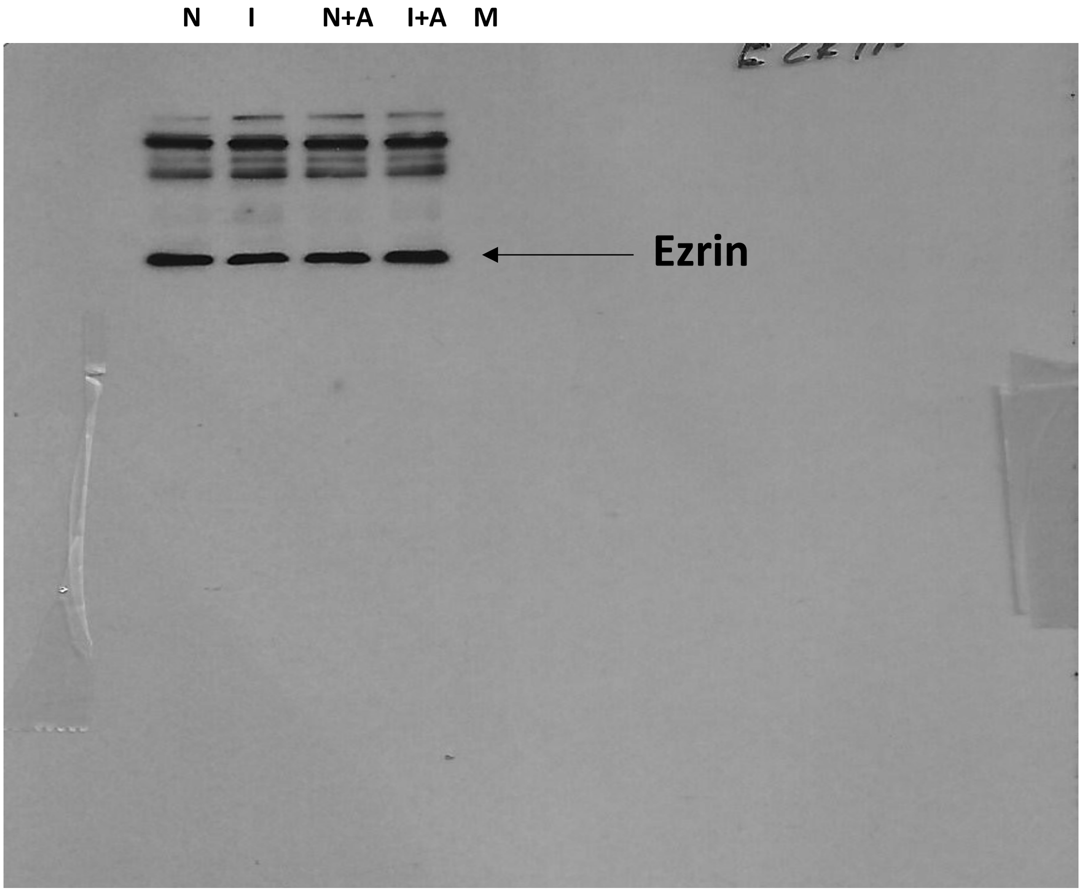

B0AT1-Experiment 2 and 3

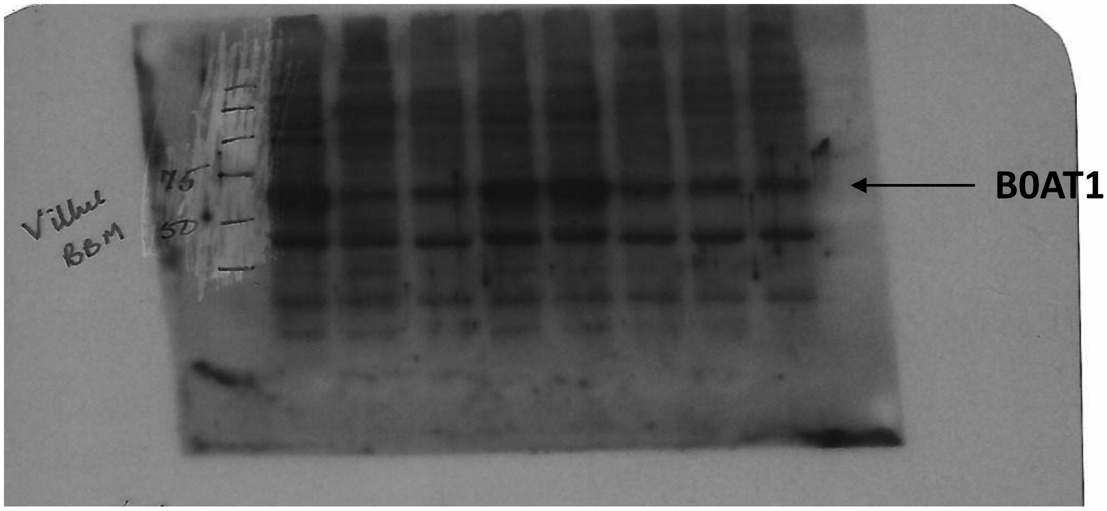

Ezrin-Experiment 2 and 3

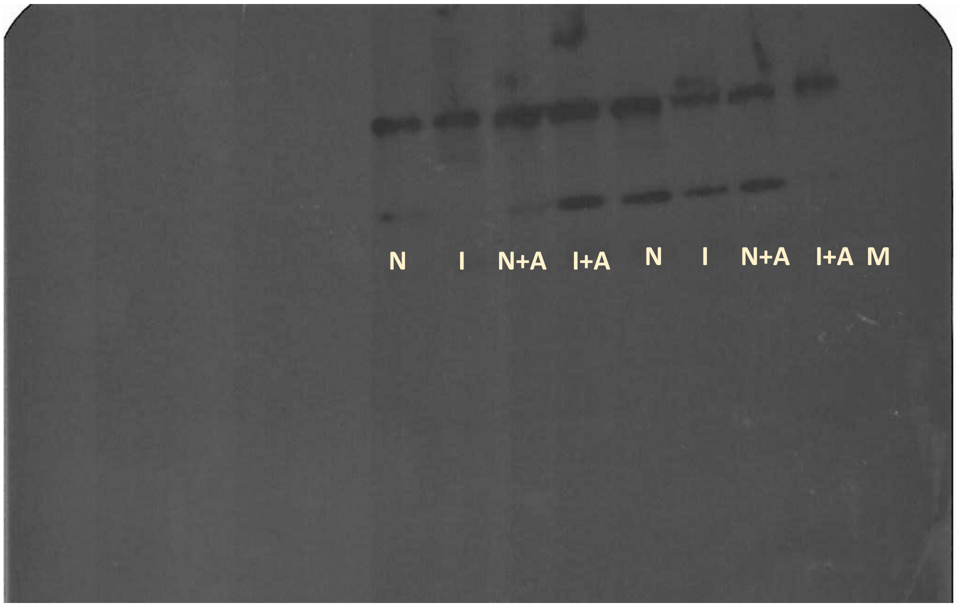

Supplement: S1 File — (PDF) [file pone.0294387.s001.pdf]
