# Supplementary figures and images for: Correction: Cyclooxygenase pathway mediates the inhibition of Na-glutamine co-transporter B0AT1 in rabbit villus cells during chronic intestinal inflammation
Source: PLoS One. 2023 Nov 9;18(11):e0294387. doi: 10.1371/journal.pone.0294387 (PMC10635497; doi:10.1371/journal.pone.0294387)

### B0AT1-Experiment 4 and 5

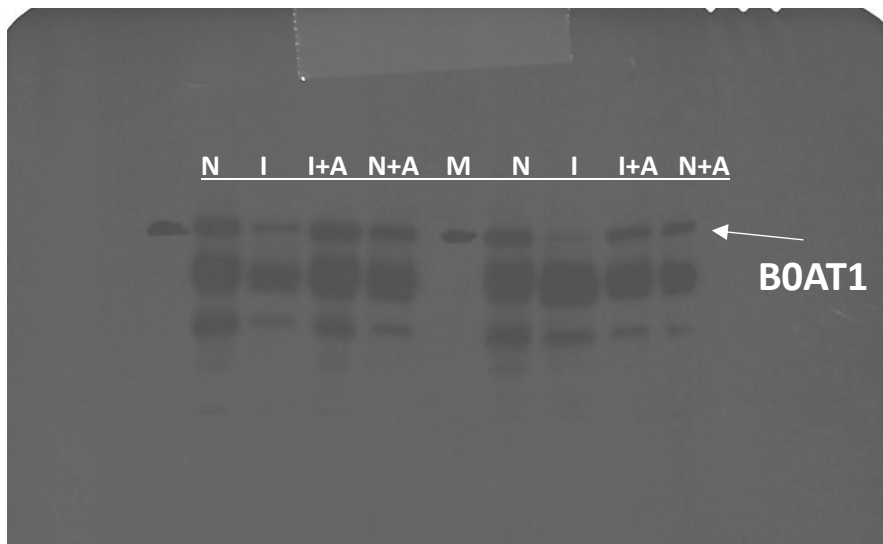

### Ezrin-Experiment 4 and 5

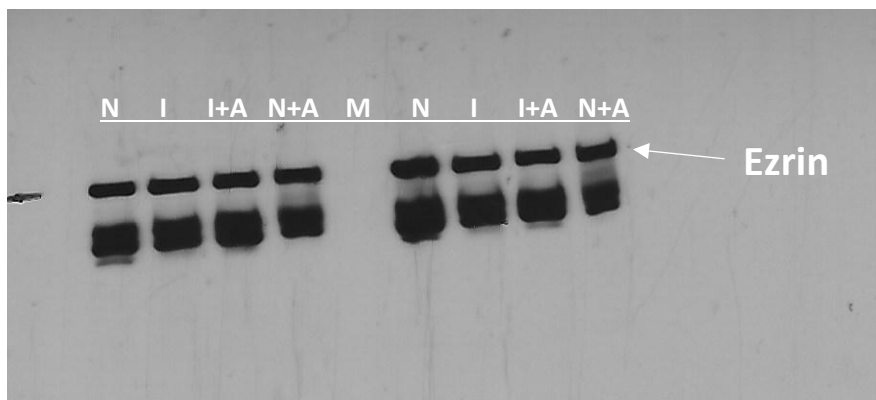

Supplement: S2 File — (PDF) [file pone.0294387.s002.pdf]
