# Supplementary material for: Correction: Cyclooxygenase pathway mediates the inhibition of Na-glutamine co-transporter B0AT1 in rabbit villus cells during chronic intestinal inflammation
Source: PLoS One. 2023 Nov 9;18(11):e0294387. doi: 10.1371/journal.pone.0294387 (PMC10635497; doi:10.1371/journal.pone.0294387)
Supplement: S3 File — (PDF) [file pone.0294387.s003.pdf]

Updated S4 Western quantitation-Experiments1-5

Experiment 1: B0AT1			Experiment 1: Ezrin			net protein/net loading	
		Net protein (after removal of background)			Net Loading (after removal of background)		
	Integrated density value	%		Integrated density value	%		
Normal	19204035	30.4	Normal	13894591	25.1	1.211155	1.00
Inflamed	7612788	12	Inflamed	14021880	25.3	0.474308	0.39
N+ATK	19085579	30.2	N+ATK	13749629	24.8	1.217742	1.01
I+ATK	17304232	27.4	I+ATK	13791563	24.9	1.100402	0.91

Experiment 2: B0AT1			Experiment 2: Ezrin				
	Integrated density value	%		Integrated density value	%		
Normal	1820936	26.9	Normal	6190952	26	1.034615	1.00
Inflamed	1207894	17.9	Inflamed	6046769	25.4	0.704724	0.68
N+ATK	1927106	28.5	N+ATK	5918602	24.9	1.144578	1.11
I+ATK	1804838	28.7	I+ATK	5610872	23.6	1.216102	1.18

Experiment 3: B0AT1			Experiment 3: Ezrin				
	Integrated density value	%		Integrated density value	%		
Normal	3993224	26.4	Normal	6186432	22.2	1.189189	1.00
Inflamed	2620552	17.3	Inflamed	7050057	25.3	0.683794	0.58
N+ATK	4101358	27.1	N+ATK	6972596	25	1.084	0.91
I+ATK	4434369	29.3	I+ATK	7641734	27.4	1.069343	0.90

Experiment 4: B0AT1			Experiment 4: Ezrin				
	Integrated density value	%		Integrated density value	%		
Normal	6396642	29.5	Normal	39876277	25.4	1.161417	1.00
Inflamed	2748838	12.7	Inflamed	38975380	24.8	0.512097	0.44
N+ATK	6095586	28.1	N+ATK	40695552	25.9	1.084942	0.93
I+ATK	6420277	29.6	I+ATK	37651054	24	1.233333	1.06

Experiment 5: B0AT1			Experiment 5: Ezrin				
	Integrated density value	%		Integrated density value	%		
Normal	5189484	32.2	Normal	35908999	25.2	1.277778	1.00
Inflamed	1727303	10.7	Inflamed	35442761	24.9	0.429719	0.34
N+ATK	4554821	28.3	N+ATK	37847762	26.6	1.06391	0.83
I+ATK	4630301	28.8	I+ATK	33020598	23.2	1.241379	0.97

All 5 Experiments

	I	N+ATK	I+ATK
	0.39	1.01	0.91
	0.68	1.11	1.18
	0.58	0.91	0.9
	0.44	0.93	1.06
	0.34	0.83	0.97
**Avg**	**0.486**	**0.958**	**1.004**
**SEM**	**0.062897**	**0.047582**	**0.052402**
